# Alterations in *Salmonella diarizonae* proteome after exposure to human serum

**DOI:** 10.3389/fcimb.2026.1875815

**Published:** 2026-10-01

**Authors:** Eva Krzyżewska-Dudek, Katarzyna Kapczyńska, Paweł Pasikowski, Bartłomiej Dudek, Jacek Rybka

**Affiliations:** 1 Department of Immunology of Infectious Diseases, Hirszfeld Institute of Immunology and Experimental Therapy, Polish Academy of Sciences, Wrocław, Poland; 2 Captor Therapeutics, Wrocław, Poland; 3 Platform for Unique Model Applications (PUMA), Division of Translational Technologies, Faculty of Pharmacy, Wrocław Medical University, Wrocław, Poland

**Keywords:** complement, O-antigen chain length, proteome, *Salmonella*, serum

## Abstract

*Salmonella* bacteria can modify structures of various cell envelope components to survive in extremely adverse environments and evade the host’s immune response. Bacterial resistance to serum is crucial for the development of systemic infections. Here, we investigated how long-term exposure to human serum alters the length of lipopolysaccharide and the outer membrane proteome shape of *Salmonella diarizonae*. Experiments have shown that prolonged exposure to human serum affects the survival rate of *S. diarizonae* but not the average length of lipopolysaccharide. Interestingly, after the analysis of preparations of outer membrane proteins we have shown, that multiple passages in human serum induced changes in the outer membrane proteome. Among the proteins with higher abundance compared to the strain prior to serum exposure, proteins described as complement resistance proteins of *Salmonella* bacteria could be identified (PagC, PgtE). Additionally, an upregulation of proteins involved in the biosynthesis of polysaccharides (RfbD, RfbA, RfbF, YaeD, PmrF, WzzE) was observed, suggesting that modifications in the structure of lipid A and modifications in the length of the enterobacterial common antigen (ECA) may be involved in the protection of *S. diarizonae* from the bactericidal action of human serum.

## Introduction

*Salmonella* bacteria are facultative intracellular pathogens, usually invading the gastrointestinal tract causing salmonellosis. Nontyphoidal *Salmonella* (NTS) strains are a major epidemiological problem, as they cause approximately 93 million cases of gastroenteritis worldwide each year (Bermudez-Aguirre et al., 2025). In the European Union NTS are among the leading causes of bacterial diarrhea. In 2024, 79 703 cases of human infections caused by NTS were recorded in European countries (European Food Safety Authority (EFSA), European Centre for Disease Prevention and Control (ECDC), The European Union One Health 2024 Zoonoses Report, 2025). It is worth noting that the true number of infections is much higher, as only 1-5% of cases are laboratory-confirmed and reported (Hoelzer et al., 2011). The gastrointestinal form of infection is more common in developed countries, while in sub-Saharan Africa NTS can lead to bloodstream infections, often without gastrointestinal symptoms (Balasubramanian et al., 2019; Ondari et al., 2019). During infection, microbes encounter the host’s immune defense mechanisms. The complement system, a tightly regulated network of proteins, plays a crucial role in innate immunity. The basic functions of the complement cascade are: labelling of foreign invaders, activation of inflammatory reactions, attraction of phagocytes, removal of immune complexes, activation of immune cells, and direct removal of microorganisms (Kemper et al., 2023). However, pathogens can develop resistance against the bactericidal action of serum, utilizing several molecular mechanisms. Bacteria are able to modify the biosynthesis and structure of cell envelope components, like lipopolysaccharide (LPS), outer membrane proteins (OMPs), fimbriae or polysaccharide capsules, to survive in an unfavorable environment (Looney and Steigbigel, 1986; Murray et al., 2003; Wilson et al., 2011; Krzyżewska-Dudek et al., 2022). LPS, as an integral component of the outer membrane of Gram-negative bacteria, covers about 70% of the cell surface (Klein and Raina, 2019). Some NTS strains produce LPS with clearly distinct fractions, differing in the number of sugar repeating units (RU) in the O-antigen part of the molecule. Low molecular weight LPS is characterized by up to 15 RU, long (L-OAg) by 16–35 RU, and very-long (VL-OAg) by more than 100 RU (Bravo et al., 2008). An appropriate density of L-OAg and VL-OAg LPS on the surface of the bacterial cell is necessary to evade the complement system. We previously demonstrated that *Salmonella* Enteritidis requires L-OAg LPS to resist complement-mediated killing (Krzyżewska-Dudek et al., 2024). The polysaccharide part of LPS physically separates the membrane attack complex (MAC) from the hydrophobic part of the cell membrane, preventing cell lysis (Joiner et al., 1982a, b, 1986; Grossman et al., 1987). *Salmonella* strains belonging to the serogroup O48 are unique and characterized by the presence of sialic acid (NeuAc) in the O-antigen part of the LPS molecule (Gamian et al., 2000). Previously, the link between *Salmonella* O48 strain LPS length and resistance to the bactericidal activity of human serum was established. Pawlak et al. demonstrated that some *Salmonella* O48 strains acquire serum resistance and increase the average LPS length after prolonged exposure to human serum (Pawlak et al., 2017).

Besides LPS, OMPs can also play a significant role in the adaptation of the bacteria to unfavorable, fluctuating environmental conditions (Achouak et al., 2001; Krzyżewska-Dudek et al., 2022). OMPs are responsible for the resistance of *Salmonella* strains to serum. Several mechanisms by which OMPs interact with the complement system are known (Krzyżewska-Dudek et al., 2022). Protein PagC recruits regulatory complement proteins, such as factor H or C4b-binding protein, to avoid a response from the immune system of the host (Nishio et al., 2005). Protein TraT inhibits the formation of a functional MAC complex (Pramoonjago et al., 1992). PgtE, an enterobacterial aspartic protease, cleaves complement components like C3b, C4b and C5 (Ramu et al., 2007; Riva et al., 2015). It has been shown that *Salmonella* O48 strains, both resistant and sensitive to serum, express certain characteristic OMPs on the cell membrane (Bugla-Płoskońska et al., 2011). Serum resistance of *S*. Hadar, *S*. Sydney, *S*. Marina and *S*. Erlangen was previously shown to be connected with the presence of OMPs in the mass range of 17–35 and 51–54 kDa (Futoma-Kołoch et al., 2012; Futoma-Kołoch et al., 2019).

The interaction between bacteria and components of the immune system creates a selective pressure for bacteria to evolve new mechanisms to evade the immune system. The resistance to human serum is a complex process in which various structures of the bacterial cell may be involved. However, data on differences in the OMPs profile of *Salmonella* O48 strains after exposure to human serum are limited. Therefore, the objective of the present study was to characterize changes in protein abundance levels after prolonged contact with human serum using quantitative proteomic analysis.

## Materials and methods

### Bacterial strain

The study was carried out on *Salmonella enterica* subsp. *diarizonae* (*S. diarizonae*) PCM 2511 from the Polish Collection of Microorganisms (PCM), Hirszfeld Institute of Immunology and Experimental Therapy, Polish Academy of Sciences, Wrocław, Poland. This strain belongs to the O48 serogroup which is characterized by the presence of NeuAc in the O-antigen region of the LPS molecule, what allows for the determination of the average LPS length by GC-MS/MS analysis (Rybka and Gamian, 2006; Bugla-Płoskońska et al., 2010). It has been shown previously that serum resistance in the O48 serogroup is connected with LPS length and changes in the outer membrane proteome (Futoma-Kołoch et al., 2012; Pawlak et al., 2017). Taking this into account, we selected the *S. diarizonae* strain as a representative of the given serological group.

### Passages in human serum

Blood samples for serum collection were obtained from the Regional Centre of Transfusion Medicine and Blood Bank in Wrocław by the Center’s employees. The samples were collected in accordance with the applicable rules set out in the Act on the public blood service (May 20, 2016) and the Directive of the European Parliament and Council (2002/98/EC, January 27, 2003). Blood samples from 108 healthy donors were collected in disposable tubes containing clot activator and separating gel, then stored at 4 °C for 1 hour to allow serum to separate from the remaining blood components. Next, the samples were centrifuged (10 min, 2 500 × g, 4 °C) and the obtained serum samples were pooled and frozen in 500 µl aliquots at -80 °C and stored for no longer than 3 months. Only the serum samples without hemolysis and lipemia were used for experiments. The complement activity in the pooled serum was measured with Human Complement C3 & C4 ‘Nl’ Bindarid Radial Immunodiffusion (The Binding site Group Ltd., United Kingdom) according to the manufacturer’s protocol.

To determine how long-term incubation in serum affects the proteome, *S. diarizonae* PCM 2511 was passaged in normal human serum (NHS) as described by Pawlak et al., 2017 with minor modifications. Briefly, bacteria were grown for 18 hours with shaking (180 rpm) at 37 °C in 50 ml lysogeny broth (LB, Sigma-Aldrich, USA). 1 ml of the overnight culture was stored in glycerol stocks for OMPs isolation, 4 ml of the culture was centrifuged (20 min, 3 500 × g, 4 °C), and the pellet was frozen at -20 °C as material for the measurement of the average LPS length. A part of the overnight culture was transferred to 5 ml LB to obtain an optical density (OD_600_) of 0.1 and grown to OD_600_ of 0.3 (180 rpm, 37 °C). In the next step, the culture was centrifuged (20 min, 3 500 × g, 4 °C) and the pellet was suspended in 3 ml phosphate-buffered saline (137 mM NaCl, 2.7 mM KCl, 8 mM Na_2_HPO_4_, and 2 mM KH_2_PO_4_ (Sigma-Aldrich, USA), pH 7.4) (PBS) and then diluted in the same saline solution to obtain a mean colony forming unit (CFU/ml) value of 10^7^. For the test, 500 µl of bacterial cell suspension was mixed with 500 µl of NHS in order to obtain a final serum concentration of 50% (50% NHS). The mixture was incubated with shaking (180 rpm) at 37 °C for 0 and 180 minutes (T_0_ and T_180_, respectively). Selected dilutions were plated in duplicate on LB agar plates and incubated at 37 °C for 24 hours to determine the average number of bacterial cells in a given serum incubation time point. After T_180_, the NHS culture was centrifuged (20 min, 3 500 × g, 4 °C), the pellet was suspended in 500 µl PBS, transferred to 50 ml fresh LB and re-cultured for 18 hours with shaking (180 rpm) at 37 °C. The entire procedure was repeated for 5 days. Survival rates were calculated as the number of bacterial cells (CFU/ml) at T_180_ versus the number at T_0_ (set as 100%). Pooled heat-inactivated serum (HIS) was used as a control (final serum concentration 50%). HIS was prepared by incubating NHS at 56 °C for at least 30 min. The experiment was performed in three biological replicates.

### Determination of the average LPS length by GC-MS/MS analysis

The measurement of the NeuAc level in comparison to the amount of Kdo (3-Deoxy-d-manno-octulosonic acid), which is constant in the LPS molecule, allows for the determination of the average length of the O-specific part of LPS (Rybka and Gamian, 2006; Bugla-Płoskońska et al., 2010; Krzyżewska-Dudek et al., 2024). Frozen bacterial pellets obtained during passages were thawed and washed three times with PBS. 500 µl of PBS was added to the pellet, shaken, and centrifuged (10 min, 3 500 × g, 4 °C). Next, 100 µl of bacterial cells were lyophilized with the addition of 20 µl of an internal standard - perseitol (1 mg/ml in Milli-Q) (Koch-Light Laboratories Ltd., UK). 1 ml of methanolic HCl solution prepared by mixing 10 ml of methanol (POCH, Poland) with 1.2 ml of acetyl chloride (Sigma-Aldrich, USA) was added to each lyophilized sample. Samples were incubated for 1 hour at 80 °C. After incubation, the tubes were cooled and evaporated under a stream of N_2_ (Air Products, USA). 100 µl of acetic anhydride (POCH, Poland) and 20 µl of pyridine (Merck, KGaA, Germany) were added to the dried test tubes and the samples were incubated for 30 minutes at 80 °C. After incubation, the tubes were re-cooled and evaporated under a stream of N_2_. The samples were then rinsed twice with methanol (POCH, Poland). 100 μl of ethyl acetate (Sigma-Aldrich, USA) was added to the dried test tubes and analyzed. The analysis was performed using gas chromatography coupled with mass spectrometry (GC-MS). The analysis was performed using a FOCUS chromatograph equipped with a Zebron ZB-5HT 30 m × 0.25 mm × 0.25 μm w/5 m Guardian (Phenomenex, Germany) column, coupled with an ITQ 700 ion trap mass detector (Thermo Scientific, USA). The sample was run in the temperature range of 150-270 °C for 16 minutes. The detected fragment of the sialic acid derivative (acetylated methyl glycoside NeuAc methyl ester) was the primary ion of m/z = 446 or the secondary of m/z = 386, while the detected fragment of Kdo derivative (acetylated methyl glycoside Kdo methyl ester) was the primary ion of m/z = 375 or secondary of m/z = 195. The obtained results were analyzed using Xcalibur software (Thermo Scientific, USA).

The measured amount of NeuAc, as a component of the O-specific polysaccharide, was compared to the amount of Kdo, as a component of the LPS core. The resulting proportions between the O-specific chain and core oligosaccharide component corresponded to the average length of the lipopolysaccharide molecule. The experiment was performed in three biological replicates.

### Isolation of outer membrane proteins

OMPs were isolated from wild-type *S. diarizonae* PCM 2511 and after five days of passaging in NHS according to Murphy and Bartos with modifications (Murphy and Bartos, 1989; Bednarz-Misa et al., 2014). Bacterial strains obtained before and after passages were grown from the prepared glycerol stocks for 18 hours with shaking at 37 °C in 25 ml LB. The cultures were centrifuged (20 min, 3 500 × g, 4 °C) and the pellets were suspended in 1.25 ml of 1M sodium acetate (POCH, Poland), 1 mM β-mercaptoethanol (Merck, USA). Next, 11.25 ml water solution containing 5% (w/v) Zwittergent Z 3–14 (Calbiochem, USA) and 0.5 M CaCl_2_ (POCH, Poland) was added. The suspended pellet was incubated on a magnetic stirrer for 1 hour at room temperature. Then, 3.13 ml of cold 96% ethyl alcohol (POCH, Poland) was added to the mixture and centrifuged (10 min, 12 000 × g, 4 °C). Subsequently, 46.75 ml of cold 96% ethyl alcohol was added very slowly to the supernatant under constant magnetic stirring, and the mixture was centrifuged (10 min, 12 000 × g, 4 °C). The resulting precipitate was dried at ambient temperature for 1 hour. Then 2.5 ml 50 mM Trizma-Base (Sigma-Aldrich, USA) buffer, pH 8.0, containing 0.05% (w/v) Zwittergent Z 3–14 and 10mM EDTA (Sigma-Aldrich, USA) was added to the pellet. After 1 hour of incubation at ambient temperature, the mixture was incubated at 4 °C overnight. Next, the mixture was centrifuged (10 min, 12 000 × g, 4 °C) and the supernatant containing OMPs was stored at -20 °C for further experiments. The concentration of the isolated proteins was determined using the commercial Pierce™ Coomassie Plus (Bradford) Assay Kit (Thermo Scientific, USA) according to the manufacturer’s procedure. The experiment was performed in three biological replicates.

### Reduction, alkylation, digestion, and TMT labeling

FASP (Filter-Aided Sample Preparation) method was used to prepare OMP-containing samples for mass spectrometric analysis (Wiśniewski et al., 2009). Briefly, 80 µg of samples were subjected to reduction, alkylation and digestion on centrifugal filters with 3 kDa molecular weight cut-off (VWR, Poland). The filter was conditioned by rinsing with 500 µl of Milli-Q water and 100 µl of 100 mM TEAB buffer (triethyl ammonium bicarbonate, Thermo Scientific, USA) by spinning the filter each time after applying the solution (15 min, 10 000 × g, 4 °C). Samples were reduced with 10 mM TCEP (tris(2-carboxyethyl)phosphine, Thermo Scientific, USA) for 1 h at 55 °C and subsequently alkylated with 17 mM iodoacetamide (Thermo Scientific, USA) in 100 mM TEAB for 30 min at ambient temperature in the dark. After incubation, the samples were centrifuged (30 min, 10 000 × g, 4 °C) and digested with trypsin solution (protein-to-enzyme ratio of 40:1 (w/w)) in 50 mM TEAB (Promega, USA) at 37 °C for 18 hours. After digestion, the peptide concentrations were determined with the Pierce™ Quantitative Colorimetric Peptide Assay (Thermo Scientific, USA). Peptides were labeled with the TMTsixplex Label Reagent Set (Thermo Scientific, USA) according to the manufacturer’s instructions. The resulting samples were stored at -80 °C until further processing. All reagents and solvents were suitable for LC-MS analysis.

### LC-MS/MS

LC-MS measurements were performed on a nanoAcquity liquid chromatograph (Waters, USA) coupled to a Q Exactive mass spectrometer (Thermo-Scientific, USA). RP-18 (80 µm × 20 mm, Waters) and nanoAcquity BEH C18 (75 µm × 250 mm, 1.7 µm, Waters) were used as pre-column – column maintained at 35 °C. The mobile phase contained water and acetonitrile with 0.1% addition of formic acid, and a two-step gradient was used (0-15% acetonitrile in 80 min, then 15-35% acetonitrile in 85 min). Flow rate was 250 nl/min, HCD fragmentation of the top 12 ions was performed (data-dependent acquisition).

### Protein identification and quantification

Mass spectra were processed with Proteome Discoverer 2.4. Sequest HT was used to extract and annotate MS/MS spectra. A database search was conducted in UniProtKB *Salmonella* Typhimurium LT2 taxonomy (accessed on 2021-07-27). The following static modifications were set as method parameters: TMT6plex/+229.163 Da (Any N-Terminus), Carbamidomethyl/+57.021 Da (C), TMT6plex/+229.163 Da (K); dynamic modification: Oxidation/+15.995 Da (M). Trypsin was used as an enzyme, with 2 allowed missed cleavage sites. Protein analysis was performed using a 10 ppm precursor mass tolerance, fragment mass tolerance was set to 0.02 Da. Only proteins with at least two high-confidence peptide matches were taken into account, and a strict parsimony rule was applied, and the false discovery rate (FDR) threshold was set to 0.01. TMT reporter ion intensities were utilized for peptide quantification with PD 2.4. Chromatograms were normalized for total peptide amount. No missing value imputation was used and the results with at least one missing value were filtered out. The experiment was performed in nested design, with samples coming from passages before passaging and after 5 days, coming from the single biological replicate. The mass spectrometry proteomics data have been deposited to the ProteomeXchange Consortium via the PRIDE (Perez-Riverol et al., 2025) partner repository with the dataset identifier PXD083351 and 10.6019/PXD083351.

The overall workflow for the proteomic data analysis of the tested *S. diarizonae* PCM 2511 strain before and after five consecutive serum exposures is shown in **Figure 1**.

**Figure 1:**
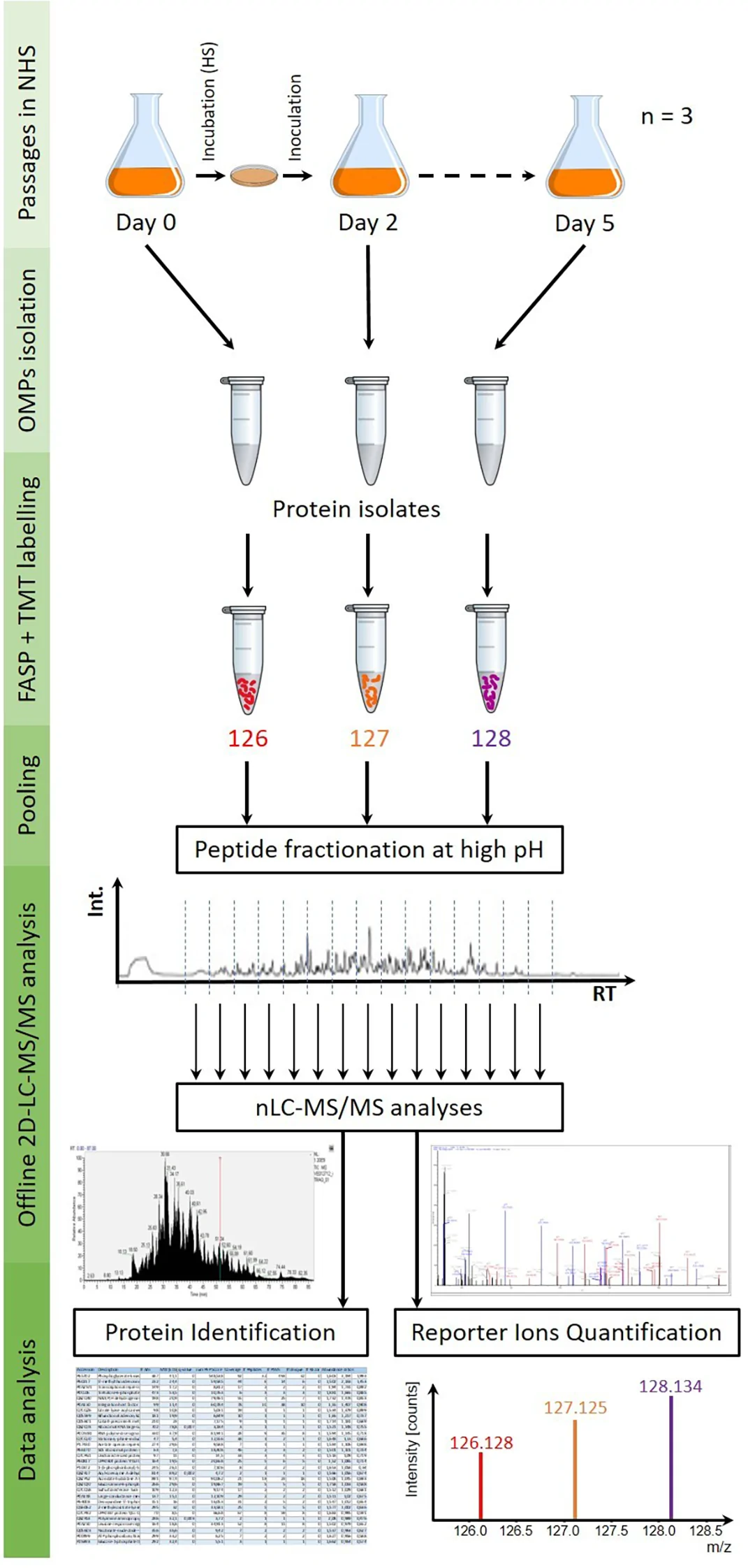
Schematic overview of the proteomic data analysis before and after five consecutive passages in human serum.

### Functional enrichment analysis

Differentially expressed proteins after exposure to human serum were subjected to gene ontology (GO) term enrichment to investigate the biological process (BP), molecular function (MF), and cell compartment (CC) using DAVID v6.8 Bioinformatics Resources (Database for Annotation, Visualization and Integrated Discovery) (Huang et al., 2009a, b). Additionally, a functional annotation clustering was performed for the upregulated proteins after five consecutive passages in serum. Gene lists of differentially expressed proteins after serum exposure were uploaded and analyzed using default settings with a minimum count threshold of 2 and an EASE score threshold of 0.1.

### Statistical analysis

All graphs were prepared and data were analyzed with GraphPad Prism (version 9.2.0 for Windows, GraphPad Software, San Diego, California, USA, www.graphpad.com). Data normality distribution was tested using the Shapiro-Wilk test. ANOVA followed by Dunnett’s multiple comparisons test was used to assess the statistical significance of the results.

## Results and discussion

### Passaging in human serum affects the survival rate of *S. diarizonae* but not the average length of lipopolysaccharide

#### Survival of bacteria in serum

In the present work, we investigated how long-term exposure to human serum affects the survival rate of *Salmonella diarizonae* PCM 2511. The serum pool used in this study exhibited normal complement activity, with a result of 1310 mg/L, which is within the manufacturer’s reference range. *S. diarizonae* PCM 2511 showed no differences in the survival rate after growth in 50% HIS (**Supplementary Figure S1**) and an increase in the survival rate after passaging in 50% NHS (**Table 1**; **Figure 2**). After the first passage, the survival rate of the tested strain was 0.05%. We observed only a slight increase in the survival rate after the following two passages: 3% on day two and 4% on day three. After the fourth passage, the survival rate in 50% NHS increased to 76% and finally reached 244% after the fifth passage (**Table 1**, **Figure 2**). Although these results alone do not allow us to determine whether the increase in survival was due to an adaptation specific to the complement factor, or was merely the result of selection during successive passages, further results point to a mechanism of bacterial adaptation.

**Table 1:** Bactericidal activity against *S. diarizonae* PCM 2511 during passages in 50% NHS.

Bactericidal activity of 50% NHS				
Day of passage	CFU/ml		Survival [%]	
	T_0_	T_180_	T_0_	T_180_
1	1,18 × 10^7^ ± 2,68 × 10^6^	5,19 × 10^3^ ± 2,08 × 10^3^	100 ± 23	0,05 ± 0,03
2	1,02 × 10^7^ ± 7,21 × 10^5^	2,9 × 10^5^ ± 7,34 × 10^4^	100 ± 7	3 ± 0,5
3	1,38 × 10^7^ ± 5,72 ×10^6^	5,58 × 10^5^ ± 2,66 × 10^5^	100 ± 41	4 ± 2
4	2,89 × 10^7^ ± 1,01 × 10^7^	1,99 × 10^7^ ± 3,02 × 10^6^	100 ± 35	76 ± 21
5	6,31 × 10^6^ ± 1,24 × 10^5^	1,54 × 10^7^ ± 4,34 × 10^6^	100 ± 2	244 ± 69

NHS, normal human serum; CFU, colony forming unit.

**Figure 2:**
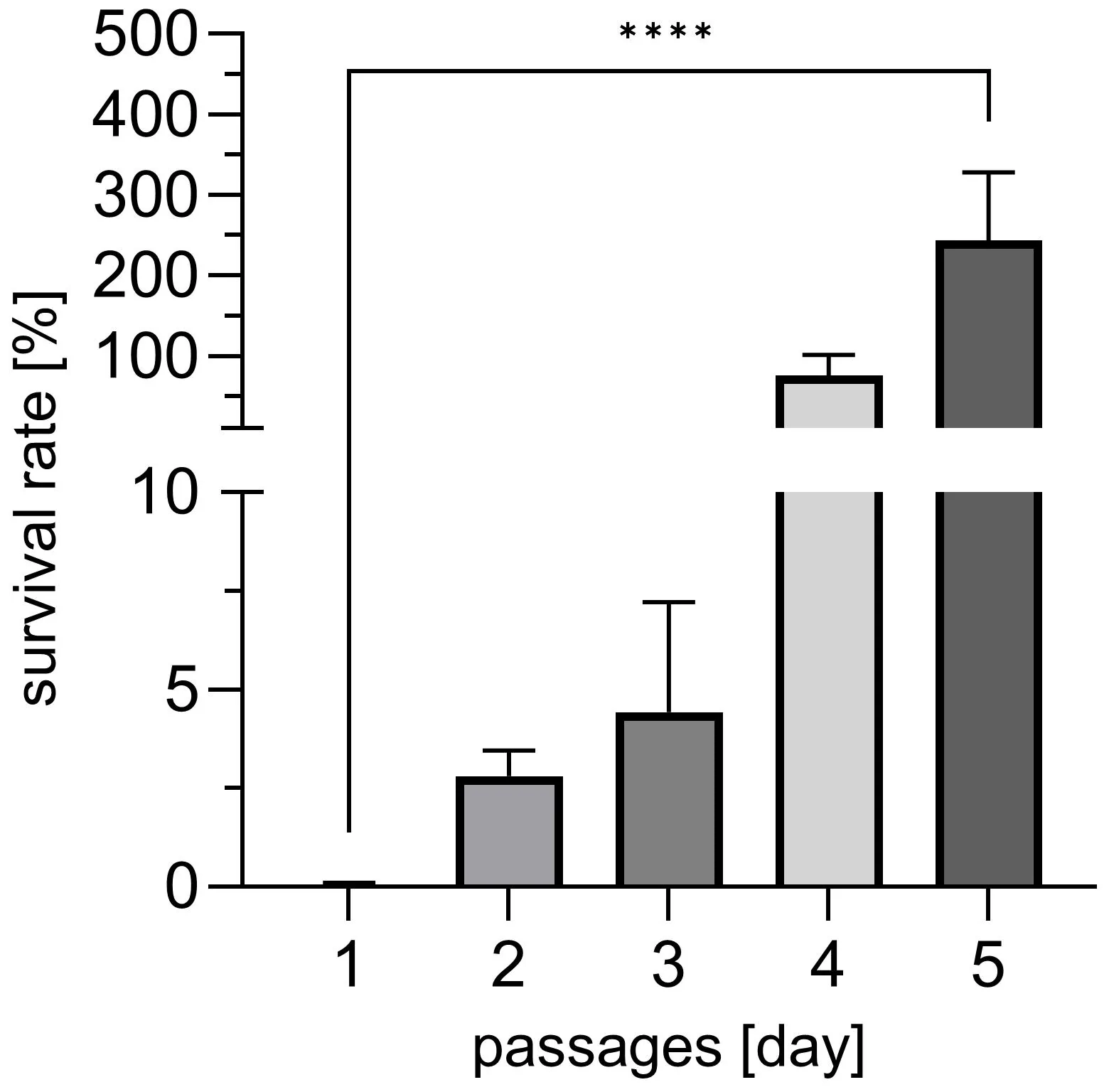
Survival rate of *S. diarizonae* PCM 2511 after passages in 50% NHS. Data are shown as mean ± SD and were analyzed by one-way ANOVA and Dunnett’s multiple comparisons. The mean of each column was compared with the mean of the column after the first day of passages in 50% human serum. Statistically significant difference is marked with asterisks (****p ≤ 0.0001).

#### LPS O-antigen length after passages

In our previous study, we demonstrated that *Salmonella* O48 strains can vary in the average LPS length, expressed as the NeuAc/Kdo ratio, after exposure to NHS (Pawlak et al., 2017). However, in that work, we measured LPS length once, after nine serum passages, and did not monitor changes in LPS length after each passage. Here, we measured the average LPS length after every passage (**Figure 3**). In contrast to serum survival, we did not observe an increase in the NeuAc/Kdo ratio after five passages in 50% NHS. After the first passage, the NeuAc/Kdo ratio reached 0.43% and dropped to 0.27% after the second. After the third day, the value of NeuAc/Kdo increased to 0.6% and decreased again to 0.33% on day four to finally reach the value of 0.09% after the fifth passage (**Figure 3**). Overall, the experiment did not show a clear tendency to extend the O-specific chain length of LPS after prolonged exposure to 50% human serum.

**Figure 3:**
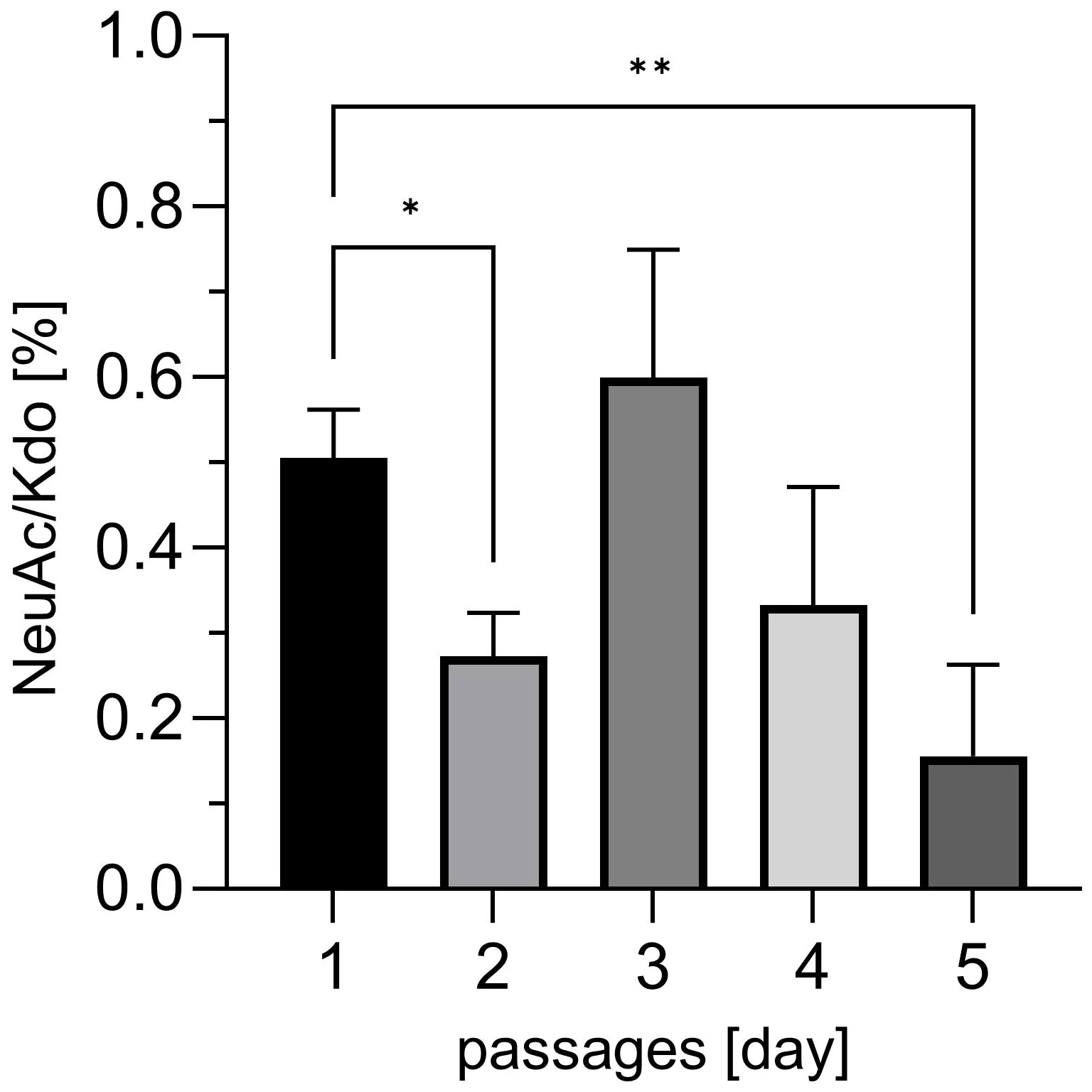
NeuAc/Kdo content (%) in *S. diarizonae* PCM 2511 after passaging in 50% NHS. Data are shown as mean ± SD and were analyzed by one-way ANOVA and Dunnett’s multiple comparisons. The mean of each column was compared with the mean of the column after the first day of passages in 50% human serum. Statistically significant differences are marked with asterisks (*p ≤ 0.1, **p ≤ 0.01).

The survival of *Salmonella* strains under unfavorable environmental conditions is linked to their adaptive capabilities. We previously showed that passaging in human serum of *Salmonella* O48 strains Isaszeg, Erlangen, Bongori and Hammonia can lead to the development of serum resistance and changes in the average O-antigen length expressed as NeuAc/Kdo ratio (Pawlak et al., 2017). In this study, the tested *Salmonella* O48 strain *S. diarizonae* PCM 2511 also gained resistance to 50% NHS during serum passages (**Figure 2**). However, after 5 consecutive exposures to 50% NHS, we did not observe any apparent increase in the average LPS length expressed as NeuAc/Kdo ratio in comparison to the wild-type strain (**Figure 3**), even when we measured the average LPS length after every passage in serum, not only before and after serum passages as in our previous work (Pawlak et al., 2017), on the contrary: after five passages the *S. diarizonae* PCM 2511 NeuAc/Kdo ratio was lower. That most likely indicates a change in the modal distribution of LPS O-antigen lengths and a reduction in the production of long LPS fractions (VL-OAg and L-OAg LPS). It is possible that the NeuAc/Kdo ratio would increase after additional passages in serum (9 passages instead of 5). It must be mentioned, however, that the fact, that bacteria did not show the signs of the productions of the longer O-Ag LPS fractions, does not exclude the possibility of other structural alterations, like e.g. acetylation of LPS molecule or other substitutions.

### Prolonged exposure to human serum affects the proteome shape of *S. diarizonae*

Proteins play an important role in protecting bacteria against the bactericidal activity of human serum; however, there is little information in the literature on changes induced in the transcriptome or proteome of bacteria after long-term exposure to serum. Ondari et al., examining *S*. Typhimurium gene expression after three hours of incubation with serum, showed no differences in the expression levels of genes encoding known serum-resistance proteins in *Salmonella* (Rck, TraT, PgtE) (Ondari et al., 2019). Instead, the main differences were detected in expression levels of genes involved in nutrient and iron uptake and assimilation, as well as in the synthesis of extracellular polysaccharides (Ondari et al., 2019).

Based on previous observations (Dudek et al., 2016; Pawlak et al., 2017), this study, in addition to LPS length characterization, focused on the characterization of the outer membrane proteome composition of *S. diarizonae* PCM 2511 after prolonged exposure to human serum. We isolated protein preparations enriched in OMPs before and after five consecutive exposures to 50% NHS and analyzed the proteomes for differences in protein abundance ratios. The isolated proteins were subjected to relative quantitative mass spectrometry. Despite using a method for protein isolation that preferentially isolates outer membrane proteins, we also identified cytoplasmic proteins in our approach (**Supplementary Table S1**).

In this analysis we have used the reference UniProtKB database of *Salmonella* Typhimurium LT2 as the primary global model organism for studying the *Salmonella* genus, which contained 1856 reviewed records. This approach allowed us to perform a more detailed analysis of proteins; there is significantly more verified information and curated protein entries for *Salmonella* Typhimurium, while proteins entries for other serovars in the database are mostly kept in the unreviewed, automatically annotated section of UniProtKB. The proteomic analysis against the reference database performed with Proteome Discoverer 2.4 (Thermo Scientific) identified 807 proteins, of which 732 had a high FDR protein Confidence level (**Supplementary Table S1**). Of these 732 proteins, 85 were upregulated and 7 downregulated after five consecutive serum exposures (fold change (FC) ≥ 1.5-fold, ≤ 0.5, P-values <0.05) (**Table 2**). The up- and downregulated proteins after five consecutive serum treatments were annotated with GO terms to characterize the roles these proteins may play in serum resistance. The proteins were classified into categories using the following GO terms: biological process (BP), cell compartment (CC) and molecular function (MF). After five passages, the upregulated proteins were clustered into 27 BP terms, 3 CC terms and 18 MF terms (**Figure 4A**). The downregulated proteins were clustered into 6 BP terms, 2 CC terms and 6 MF terms (**Figure 4B**). Additionally, upregulated proteins were analyzed with the functional annotation clustering tool. The analysis revealed 5 clusters with an enrichment score greater than 0.80 (**Table 3**). To reduce redundancy and improve biological interpretation, we grouped similar biological annotations and genes. The functional annotation clustering of upregulated proteins after long-term serum exposure identified proteins involved in the biosynthesis of polysaccharides, amino-acid biosynthesis, biosynthesis of co-factors and fatty acid/lipid metabolism (**Table 3**).

**Table 2:** Differentially expressed proteins of *S*. *diarizonae* after five consecutive exposures to 50% NHS.

Proteins	Accession	Description	Gene symbol	Abundance ratio: 5 days / WT
Upregulated	P22036	Magnesium-transporting ATPase. P-type 1	*mgtB*	2.27
	Q8ZRT5	GMP reductase	*guaC*	2.26
	O52324	Undecaprenyl-phosphate 4-deoxy-4-formamido-L-arabinose transferase	*pmrF*	2.18
	P64437	Uncharacterized protein YbgS	*ybgS*	2.10
	P0A1C7	Propanediol utilization protein PduA	*pduA*	2.10
	Q8ZRS3	Polyamine aminopropyltransferase	*speE*	2.08
	P40861	Gamma-glutamyl phosphate reductase	*proA*	1.99
	Q9X6M5	Guanylate kinase	*Gmk*	1.98
	P26396	Glucose-1-phosphate cytidylyltransferase	*rfbF*	1.96
	P0A2M1	Transcriptional repressor NrdR	*nrdR; ybaD*	1.94
	P37722	Esterase FrsA	*frsA; yafA*	1.92
	P0CL06	Trehalose-6-phosphate synthase	*otsA*	1.88
	P23988	Virulence membrane protein PagC	*pagC*	1.84
	P36556	Transcriptional regulatory protein BasR	*basR*	1.82
	P0AA19	DNA-binding dual transcriptional regulator OmpR	*ompR*	1.77
	P58580	Glutathione synthetase	*gshB*	1.77
	P10369	Histidinol-phosphate aminotransferase	*hisC*	1.77
	Q7CR52	Sigma factor-binding protein Crl	*Crl*	1.77
	Q8ZQX7	Glucosamine-6-phosphate deaminase	*nagB*	1.76
	Q8ZS17	UPF0246 protein YaaA	*yaaA*	1.76
	Q8ZQ40	NAD(P)H dehydrogenase (quinone)	*wraB*	1.73
	P64222	Aminomethyltransferase	*gcvT*	1.71
	Q05601	Cobalt-precorrin-8 methylmutase	*cbiC*	1.71
	Q7CPB2	UPF0337 protein YjbJ	*yjbJ*	1.68
	Q8ZN89	DNA ligase	*ligA; lig*	1.67
	O52765	Histidine--tRNA ligase	*hisS*	1.67
	Q8ZN40	Cysteine desulfurase IscS	*nifS*	1.67
	Q8ZNW0	Pyruvate kinase II	*pykA*	1.67
	Q7CPP9	Phosphoglucosamine mutase	*glmM; mrsA*	1.66
	P26393	Glucose-1-phosphate thymidylyltransferase	*rfbA*	1.66
	Q8ZKX0	Bifunctional protein GlmU	*glmU*	1.65
	P10372	1-(5-phosphoribosyl)-5-[(5-phosphoribosylamino)methylideneamino] imidazole-4-carboxamide isomerase	*hisA*	1.65
	Q05599	Bifunctional adenosylcobalamin biosynthesis protein CobU	*cobU*	1.65
	Q8ZPV2	Succinylornithine transaminase	*argD; astC*	1.65
	Q7CQJ0	Stationary-phase-induced ribosome-associated protein	*rpsV*	1.65
	Q7CQ10	HTH-type transcriptional regulator IscR	*yfhP*	1.64
	Q8ZLX7	Putrescine aminotransferase	*oat*	1.63
	P67714	Nucleoid-associated protein YejK	*yejK*	1.63
	P00499	ATP phosphoribosyltransferase	*hisG*	1.63
	O85139	3-oxoacyl-[acyl-carrier-protein] synthase 3	*fabH*	1.62
	Q8ZQU3	Succinate dehydrogenase flavoprotein subunit	*sdhA*	1.62
	P0A1V6	Ribose-phosphate pyrophosphokinase	*prsA*	1.61
	Q8ZRM8	D-glycero-beta-D-manno-heptose-1.7-bisphosphate 7-phosphatase	*yaeD*	1.61
	P65702	Phosphoglycerate kinase	*Pgk*	1.60
	Q8ZNN5	Iron-sulfur cluster carrier protein	*Mrp*	1.60
	Q8ZN83	Transaldolase A	*talA*	1.59
	D0ZVL4	RNA polymerase sigma factor RpoS	*rpoS*	1.59
	P64068	Probable GTP-binding protein EngB	*engB; yihA*	1.59
	P0A1F0	D-alanine--D-alanine ligase A	*ddl; ddlA*	1.59
	P17430	Acetate operon repressor	*iclR*	1.58
	Q7CPD4	Xaa-Pro dipeptidase	*pepQ*	1.58
	Q56062	2-methylisocitrate lyase	*prpB*	1.58
	Q8ZRJ7	Acyl-coenzyme A dehydrogenase	*fadE; yafH*	1.57
	Q7CPA2	Divalent-cation tolerance protein CutA	*cutA*	1.57
	Q9L6M2	3'-5' ssDNA/RNA exonuclease TatD	*yigW*	1.56
	O33789	ECA polysaccharide chain length modulation protein	*wzzE*	1.56
	Q8ZR68	Cysteine--tRNA ligase	*cysS*	1.56
	P60084	Protein Syd	*Syd*	1.56
	P67073	Uracil-DNA glycosylase	*Ung*	1.56
	P0A1S0	Integration host factor subunit alpha	*ihfA; himA*	1.55
	Q8ZRD8	Queuine tRNA-ribosyltransferase	*tgt*	1.55
	P0A2G9	Cobalt-precorrin-4 C(11)-methyltransferase	*cbiF*	1.55
	P64008	Deoxyuridine 5'-triphosphate nucleotidohydrolase	*dut*	1.55
	P67366	UPF0227 protein YcfP	*ycfP*	1.55
	Q05603	Nicotinate-nucleotide--dimethylbenzimidazole phosphoribosyltransferase	*cobT*	1.54
	P37419	2-amino-3-ketobutyrate coenzyme A ligase	*Kbl*	1.54
	P06185	Outer membrane protease E	*pgtE*	1.53
	Q7CQZ6	Citrate lyase acyl carrier protein 1	*citD*	1.53
	P26392	dTDP-4-dehydrorhamnose reductase	*rfbD*	1.53
	Q8ZRU8	Ribosomal RNA small subunit methyltransferase H	*mraW; yabC*	1.52
	Q8ZK67	Fructose-1.6-bisphosphatase class 1	*Fbp*	1.52
	Q8ZQ73	Ribosomal RNA large subunit methyltransferase K/L	*rlmL; ycbY*	1.52
	P60817	UPF0304 protein YfbU	*yfbU*	1.52
	Q8ZNT7	D-cysteine desulfhydrase	*yedO*	1.52
	Q7CPG1	Uncharacterized protein YicS	*STM3774*	1.52
	P39434	Soluble lytic murein transglycosylase	*Slt*	1.52
	Q8ZMD3	NADPH-dependent 7-cyano-7-deazaguanine reductase	*queF; yqcD*	1.52
	P08007	Oligopeptide transport ATP-binding protein OppF	*oppF*	1.51
	Q7CQS8	Sulfurtransferase TusE	*yccK*	1.51
	P0A1X8	Large-conductance mechanosensitive channel	*mscL*	1.51
	Q8ZP52	Aconitate hydratase A	*acnA*	1.51
	P60217	5'-methylthioadenosine/S-adenosylhomocysteine nucleosidase	*Pfs*	1.50
	P0A2S0	Leucine-responsive regulatory protein	*Lrp*	1.50
	P66170	50S ribosomal protein L29	*rpmC*	1.50
	P0A2H7	3-ketoacyl-CoA thiolase	*fadA*	1.50
Downregulated	O31042	Propanediol dehydratase small subunit	*pduE*	0.49
	P0A263	Outer membrane porin C	*ompC*	0.47
	Q7CQV9	DNA protection during starvation protein	*Dps*	0.47
	P0A1P6	Glutamine synthetase	*glnA*	0.47
	D0ZXQ1	Outer membrane porin protein OmpD	*nmpC*	0.45
	P67286	UPF0145 protein YbjQ	*orfB*	0.39
	P60659	Biosynthetic arginine decarboxylase	*speA*	0.39

**Figure 4:**
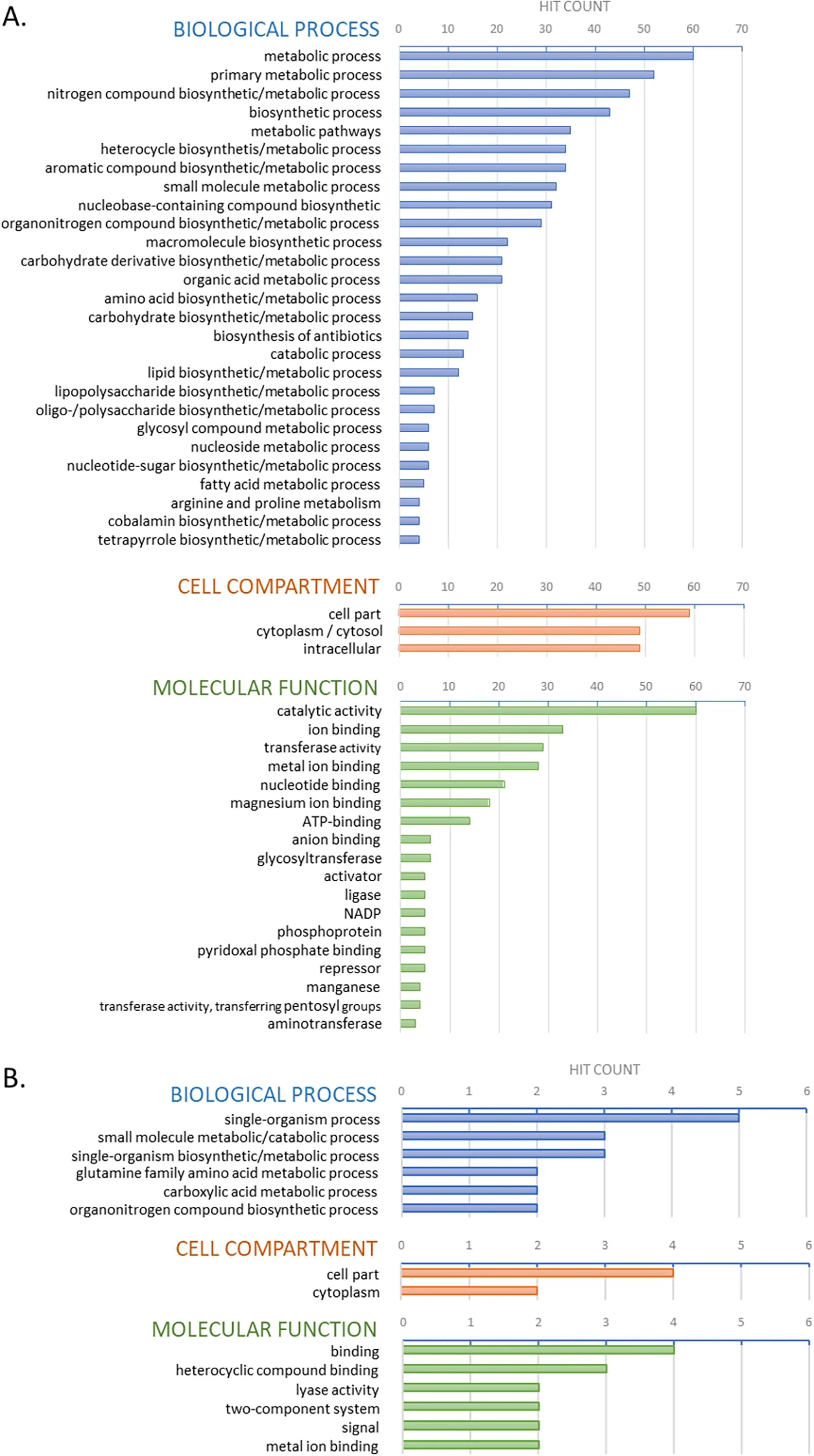
Comparison of the biological process, cell compartment and molecular function of the proteins significantly up- **(A)** and downregulated **(B)** in *S. diarizonae* PCM 2511 after five consecutive days of 50% NHS exposure. The functional analysis was performed using DAVID web service (date of access 19.09.2025) with the following parameters: tool – Functional Annotation Chart; Functional Categories – UP_KEYWORD, UP_SEQ_FEATURE; Gene Ontology – GOTERM_BP_ALL, GOTERM_CC_ALL, GOTERM_MF_ALL; Pathways – KEGG_PATHWAYS; Protein Domains – INTERPRO and filtered for a minimal count of 3 and EASE parameter of 0.5. The list was further manually curated by combining synonymic and highly similar records.

**Table 3:** Functional annotation clustering of upregulated proteins after five consecutive days of 50% NHS exposure.

Annotation cluster 1	Enrichment score: 1.89	Count	P-value
INTERPRO UP_KW_LIGAND UP_KW_MOLECULAR_FUNCTION GOTERM_MF_DIRECT	PyrdxIP-dep Trfase small PyrdxIP-dep Trfase major Pyridoxal phosphate Aminotransferase Pyridoxal phosphate binding	5 5 6 4 5	4.4E-3 7.4E-3 1.9E-2 2.7E-2 3.2E-2
Annotation Cluster 2	Enrichment score: 1.48	Count	P-value
KEGG_PATHWAY	Biosynthesis of various nucleotide sugars Amino sugar and nucleotide sugar metabolism	6 5	3.6E-3 3.7E-2
INTERPRO UP_KW_BIOLOGICAL_PROCESS	Nucleotide-diphossugar trans Lipopolysaccharide biosynthesis	4 5	1.6E-2 7.1E-2
GOTERM_BP_DIRECT UP_KW_MOLECULAR_FUNCTION	O antigen biosynthetic process Nucleotidyltransferase	3 4	7.1E-2 2.8E-1
Annotation Cluster 3	Enrichment score: 1.37	Count	P-value
GOTERM_BP_DIRECT UP_KW_BIOLOGICAL_PROCESS KEGG_PATHWAY	L-histidine biosynthetic process Histidine biosynthesis Amino-acid biosynthesis Histidine metabolism	3 3 6 3	2.4E-2 3.0E-2 1.1E-1 4.0E-2
Annotation Cluster 4	Enrichment score: 1.1	Count	P-value
GOTERM_BP_DIRECT UP_KW_BIOLOGICAL_PROCESS KEGG_PATHWAY	Cobalamin biosynthetic process Cobalamin biosynthesis Porphyrin metabolism Biosynthesis of cofactors	4 4 4 7	3.0E-2 4.1E-2 9.5E-2 3.3E-1
Annotation Cluster 5	Enrichment score: 0.85	Count	P-value
UP_KW_BIOLOGICAL_PROCESS KEGG_PATHWAY	Fatty acid metabolism Lipid metabolism Fatty acid metabolism	3 4 3	1.0E-1 2.2E-1 1.2E-1

#### Serum resistance proteins

So far, several outer membrane proteins that determine serum resistance in *Salmonella* have been identified: TraT, Rck, PagC, PgtE (Rhen and Sukupolvi, 1988; Pramoonjago et al., 1992; Nishio et al., 2005; Ramu et al., 2007; Ho et al., 2010; Riva et al., 2015). Dudek et al. showed that *S*. Enteritidis strains produce VL-OAg LPS regardless of their level of sensitivity to human serum, but differ in the composition of their outer membrane proteomes. The comparison of *S.* Enteritidis strains sensitive and resistant to the action of 75% NHS showed that PgtE protein was present in greater amounts in resistant strains, whereas OmpA was more abundant in strains sensitive to human serum (Dudek et al., 2016).

In the present study, we analyzed the proteome of *S. diarizonae* PCM 2511 after 5 consecutive exposures to human serum, focusing on proteins previously reported to be involved in complement resistance of *Salmonella*. After 5 passages in 50% NHS, PagC and PgtE proteins showed a higher abundance ratio compared with the strain before exposure to human serum (**Table 2**, upregulated). As proteins PagC and PgtE are encoded on the chromosome, while TraT and Rck are encoded on virulence plasmids (Krzyżewska-Dudek et al., 2022), it might be that the tested strain did not carry a virulence plasmid encoding those proteins. Nishio et al. showed that for *S*. Choleraesuis, the survival rate in porcine serum of a mutant without the O-antigen part and a reduced core oligosaccharide in the LPS molecule was lower in comparison to the wild-type strain, despite a similar expression level of *pagC* (Nishio et al., 2005). In contrast to PagC, the activity of PgtE is strictly connected to the length of the LPS molecule. It has been shown previously that PgtE requires short-chain LPS on the bacterial outer membrane for full activity (Kukkonen et al., 2004; Lähteenmäki et al., 2005; Ramu et al., 2007). Lähteenmäki et al. demonstrated that *Salmonella* released from macrophages produce rough LPS and exhibit increased amounts of PgtE on the cell surface, which cleaves complement proteins and protects the bacteria from cell lysis (Lähteenmäki et al., 2005). Ramu et al. showed that *S*. Typhimurium equipped with a longer type of LPS was not able to cleave complement proteins C3b, C4b and C5 in comparison to the strain characterized by short-chain LPS (Ramu et al., 2007). The results obtained in the present work show the upregulation of PgtE and PagC (**Table 2**, upregulated), which indicate that also in these bacteria they are important components of the bacterial mechanisms that enable the evasion of the bactericidal action of serum.

#### Outer membrane proteins

In the present study, outer membrane porins OmpC and OmpD were identified among the proteins with lower abundance ratios in contrast to the strain before exposure to human serum (**Table 2**, downregulated). Futoma-Kołoch et al. reported that serum resistance in *Salmonella* O48 serovars Hadar, Sydney, Marina and Erlangen might be connected with the presence of OMPs in the range of 17–, 25–, 35– and 51–54 kDa (Futoma-Kołoch et al., 2012; Futoma-Kołoch et al., 2019) and that in serovars Ngozi and Isaszeg the C3 serum protein activation is dependent on the presence of OMPs in the range of 35–48 kDa (Futoma-Kołoch et al., 2015).

#### LPS biosynthesis proteins

Notably, proteins involved in the LPS biosynthesis (RfbD, RfbA, RfbF, YaeD, PmrF) were detected among the upregulated proteins (**Table 3**, Annotation Cluster 2). While RfbD, RfbA and RfbF are proteins mainly responsible for the synthesis of the O-antigen part of the LPS molecule, protein PmrF modifies the lipid A structure with L-arabinose 4-phosphate groups influencing the surface net charge of the outer membrane. This modification masks the negative charge of lipid A and of the outer membrane, which can lead to decreased binding of cationic antimicrobial peptides but also complement components (Ernst et al., 2009; Cheng et al., 2010).

#### Enterobacterial common antigen biosynthesis protein

In addition to LPS, the enterobacterial common antigen (ECA) also plays a crucial role in maintaining the outer membrane integrity in Gram-negative bacteria. In our study, among the upregulated proteins, WzzE, responsible for chain length modulation of ECA, was identified (**Table 2**, upregulated). It has previously been shown that ECA plays a role in resistance to external stressors like antibiotics, detergents, bile salts and human serum (reviewed in (Rai and Mitchell, 2020)). Phan et al. showed that in *Escherichia coli*, mutations in five ECA biosynthesis genes (*wecA*, *wzzE, wecD, wecE, wecF*) influenced the serum resistance, while three of these genes (*wecA, wzzE, wecD*) affected the LPS structure (Phan et al., 2013). The obtained results suggest that modifications in the outer membrane shape could involve the changes in lipid A structure and ECA length. It can be hypothesized that, in addition to PgtE and PagC, the bacterium uses these mechanisms to evade the bactericidal action of human serum, which is a possibility that will be verified in further studies.

#### Fatty acids metabolism proteins

Interestingly, in our study proteins involved in fatty acid metabolism were also identified (Annotation Cluster 5: YafH, FabH, FadA; **Table 3**). Bacteria can regulate the composition of the cell membrane in response to environmental stress in order to optimize membrane fluidity and related functions (Zhang and Rock, 2008). It has been shown that changes in the fatty acid composition of the membrane can be influenced by factors such as temperature, pH, and osmolarity (Suutari and Laakso, 1994; Alvarez-Ordóñez et al., 2008). Hines et al. demonstrated that bacteria can incorporate serum-derived fatty acids into their own lipids and alter the nature of the cell surface, which may affect interactions with the host (Hines et al., 2020).

#### Proteins associated with environmental stress

The functional annotation clustering analysis did not directly reveal a cluster associated with environmental stress. However, among the 85 proteins with the highest increase in abundance ratio compared to the strain before serum exposure, proteins associated with the response to environmental stress could be identified (e.g., YjbJ, YbgS, GshB, Crl, RpoS; **Table 2**, upregulated). Alternative sigma factors play a key role in protecting bacteria from environmental stress. Their role is to regulate the expression of regulons whose gene products are responsible for adapting the bacterial cell to unfavorable environmental conditions. Xiao et al. demonstrated that in *Edwardsiella tarda*, protein RpoS was crucial for resistance and initial replication in serum (Xiao et al., 2009), indicating a clear role for this protein in stress adaptation.

### Final remarks

The predisposition of *Salmonella* to modulate the outer membrane appears essential for survival in unfavorable environments. The combined data show that NTS employ multiple strategies and mechanisms to ensure serum resistance. The proteome analysis of *Salmonella* O48 bacteria subjected to long-term serum stress reveals changes in the bacterial protein composition, which can affect bacteria’s ability to adapt to this unfavorable environment. However, it should be emphasized that the obtained proteomic data require additional validation; as with most high-throughput assay systems, certain phenomena, such as co-isolation and co-fragmentation of labeled peptides, may directly impact quantitative results. It must also be mentioned that the MS database search was performed against *S.* Typhimurium LT2, as this is the most studied and well-defined *Salmonella* serotype with the largest number of records in the UniProtKB database and serves as the primary global model organism for studying the *Salmonella* genus. Thanks to this approach, it was possible to perform much more detailed analysis of mass spectrometric results, although it must be kept in mind that the database used was not specifically created for the *S. diarizonae* proteome, and that approach may have introduced interference in the results obtained. Additionally, the presence of proteins of cytoplasmic origin in the protein preparation indicates that during the isolation method, some other proteins also co-isolated along with outer membrane proteins, which can constitute an additional limitation to the interpretation of the presented results. Nevertheless, the approach used in this study, involving the analysis of the preparations enriched in outer membrane proteins of strains subjected to human serum stress, provides a basis for further investigation of proteins and mechanisms essential for *Salmonella* O48 survival in serum, including changes in the structure of Lipid A and length of the ECA antigen.

## Data Availability

The datasets presented in this study can be found in online repositories. The names of the repository/repositories and accession number(s) can be found below: http://www.proteomexchange.org/ PXD083351.
