# Supplementary material for: Alterations in Salmonella diarizonae proteome after exposure to human serum
Source: Front Cell Infect Microbiol. 2026 Oct 1;16:1875815. doi: 10.3389/fcimb.2026.1875815 (PMC13668741; doi:10.3389/fcimb.2026.1875815)
Supplement: Supplementary file 1 [file Table2.docx]

Supplementary Figure 1. Survival rate of *S. diarizonae* PCM 2511 after passages in 50% HIS. Data are shown as means with ±SD and were analyzed by one-way ANOVA and Dunnett’s multiple comparisons. The mean of each column was compared with the mean of the column after the first day of passages in 50% HIS.
